# NRF2-mediated signaling is a master regulator of transcription factors in bovine granulosa cells under oxidative stress condition

**DOI:** 10.1007/s00441-021-03445-4

**Published:** 2021-05-19

**Authors:** Mohamed Omar Taqi, Mohammed Saeed-Zidane, Samuel Gebremedhn, Dessie Salilew-Wondim, Ernst Tholen, Christiane Neuhoff, Michael Hoelker, Karl Schellander, Dawit Tesfaye

**Affiliations:** 1 Institute of Animal Science, Animal Breeding and Husbandry Group, University of Bonn, Bonn, Germany; 2 Central Laboratory for Agricultural Climate, Agricultural Research Center, Giza, Egypt; 3 Institute of Animal Breeding and Husbandry, Animal Breeding and Genetics Group, University of Kiel, Kiel, Germany; 4 Teaching and Research Station Frankenforst, University of Bonn, Koenigswinter, Germany; 5 Department of Biomedical Sciences, Animal Reproduction and Biotechnology Laboratory (ARBL), Colorado State University, Fort Collins, CO USA

**Keywords:** Transcription factors, Granulosa cells, Oxidative stress, Apoptosis and differentiation, NRF2 signaling

## Abstract

Transcription factors (TFs) are known to be involved in regulating the expression of several classes of genes during folliculogenesis. However, the regulatory role of TFs during oxidative stress (OS) is not fully understood. The current study was aimed to investigate the regulation of the TFs in bovine granulosa cells (bGCs) during exposure to OS induced by H_2_O_2_ in vitro. For this, bGCs derived from ovarian follicles were cultured in vitro till their confluency and then treated with H_2_O_2_ for 40 min. Twenty-four hours later, cells were subjected to various phenotypic and gene expression analyses for genes related to TFs, endoplasmic reticulum stress, apoptosis, cell proliferation, and differentiation markers. The bGCs exhibited higher reactive oxygen species accumulation, DNA fragmentation, and endoplasmic reticulum stress accompanied by reduction of mitochondrial activity after exposure to OS. In addition, higher lipid accumulation and lower cell proliferation were noticed in H_2_O_2_-challenged cells. The mRNA level of TFs including *NRF2*, *E2F1*, *KLF6*, *KLF9*, *FOS*, *SREBF1*, *SREBF2*, and *NOTCH1* was increased in H_2_O_2_-treated cells compared with non-treated controls. However, the expression level of *KLF4* and its downstream gene, *CCNB1*, were downregulated in the H_2_O_2_-challenged group. Moreover, targeted inhibition of *NRF2* using small interference RNA resulted in reduced expression of *KLF9*,* FOS*, *SREBF2*, and *NOTCH1* genes, while the expression of *KLF4* was upregulated. Taken together, bovine granulosa cells exposed to OS exhibited differential expression of various transcription factors, which are mediated by the NRF2 signaling pathway.

**Supplementary Information:**

The online version contains supplementary material available at 10.1007/s00441-021-03445-4.

## Introduction

In the mammalian follicle, the oocyte is surrounded by multiple layers of somatic cells including granulosa cells, which play a significant role during oocyte maturation by producing paracrine factors including estradiol and progesterone (Wen et al. 2010). Thus, disruption of granulosa cell functions may result in poor oocyte quality and subsequently reduction in pregnancy rate (Lai et al. 2018). Previous studies have evidenced that exposure of granulosa cells to oxidative stress has led to impaired function and subsequently poor quantity and quality of oocytes (Jancar et al. 2007; Lai et al. 2018; Rajani et al. 2012). Despite the fact that accumulation of reactive oxygen species (ROS)-induced oxidative stress harms granulosa cell function, elevated levels of ROS at the time of ovulation play an indispensable role in facilitating the ovulation process (Tanabe et al. 2015).

ROS are endogenously produced during metabolic pathways as a byproduct from different cellular compartments including the plasma membrane, mitochondria, endoplasmic reticulum, and peroxisomes (Di Meo et al. 2016). Cells are equipped with a well-balanced system including antioxidant machinery to eliminate the excessive levels of ROS and maintain cellular redox homeostasis (He et al. 2017). However, disruption of this balanced system due to overwhelmed cellular ROS production leads to oxidative stress (Agarwal et al. 2006). Excessive ROS accumulation and subsequent oxidative stress could also arise from external and/or internal stressors such as heat stress (Alemu et al. 2018), inflammation (Mittal et al. 2014), and endoplasmic reticulum stress (Liu et al. 2018). Exposure of cells to oxidative stress has a negative impact on cell membrane permeability and mitochondrial activity and causes DNA damage (Kadenbach et al. 2004; Zhang et al. 2016) resulting in cell cycle arrest (Saeed-Zidane et al. 2017) and apoptosis (Sohel et al. 2019).

Mammalian cells respond to intra and extracellular signals by altering several transcription factors (TFs) that activate and/or inhibit genes involved in various biological functions (Leite et al. 2017). Some of those TFs are believed to be involved in cellular defense mechanisms against oxidative stress (Mikaeili et al. 2016; Weng et al. 2016). In bovine embryos, the capability of in vitro produced embryos to survive under oxidative stress conditions relies on the activation of nuclear factor (erythroid-derived 2)-like 2 (*NRF2* or *NFE2L2*) transcription factor at mRNA and protein levels (Amin et al. 2014). Similarly, *NRF2* is responsible for the activation of cellular antioxidant machinery in bovine granulosa cells under oxidative stress conditions (Alemu et al. 2018; Khadrawy et al. 2019; Sohel et al. 2019, 2018). Moreover, *NRF2* could be extracellularly released via exosomes and horizontally transferred to neighboring cells as a means of inducing adaptive response against oxidative stress in recipient cells (Saeed-Zidane et al. 2017).

NRF2 might particularly be involved in the regulation of other TF networks such as *NOTCH* (Zhao et al. 2016), Krüppel-like factor (*KLFs*) family members (Jang et al. 2014; Zucker et al. 2014), and sterol regulatory element-binding transcription factor (*SREBFs*) family (Amin et al. 2014; Kamisako et al. 2014), which are involved in cell proliferation, differentiation, apoptosis (Andersson et al. 2011; Miao et al. 2017), fatty acids, and cholesterol biosynthesis (Daemen et al. 2013; Shimano 2001). Some of those TFs significantly contribute to the regulation of ovarian functions (Murta et al. 2015; Natesampillai et al. 2008) such as regulation of genes involved in steroidogenesis (Liu et al. 2009). Furthermore, the overexpression of the KLF family showed a negative effect on the expression of CYP11A1 in granulosa cells (Natesampillai et al. 2008). However, increasing intracellular ROS accumulation accompanied by overexpression of *NRF2* resulted in promoting cell death via regulation of *KLF9* (Zucker et al. 2014). Moreover, apoptosis of granulosa cells could be induced via regulation of FOS after exposure to mycotoxin (Guerrero-Netro et al. 2015), which is one of the activator protein-1 (AP-1) members (Dias et al. 2013). However, little is known regarding the regulation of granulosa cell functions via various TFs under oxidative stress conditions and their relevance with the NRF2 signaling pathway. Therefore, the current study aimed to investigate the expression patterns of stress-induced TFs and their potential regulation by the *NRF2* signaling pathway in bovine granulosa cells exposed to oxidative stress.

## Materials and methods

### Experimental setup

To investigate the effect of H_2_O_2_ on the expression of the stress-related TFs and their association with the *NRF2* signaling pathway in bovine granulosa cells, two independent experiments were conducted. In the first experiment, sub-confluent bovine granulosa cells were treated with 5 µM H_2_O_2_ for 40 min as optimized previously (Saeed-Zidane et al. 2017) to induce moderate oxidative stress. Moreover, based on previous pieces of evidence and in silico analysis, the second experiment was conducted to figure out the cross-talk between *NRF2* and other candidate TFs. For that, sub-confluent bovine granulosa cells were transfected with 200 nM small interference RNA (siRNA) targeting *NRF2*. In both experiments, the cells were incubated at 37 °C in 5% CO_2_ for another 24 h after the treatment and then subjected to various phenotypic and genotypic analyses.

## In vitro culture of bovine granulosa cells

Bovine ovaries were obtained from a local abattoir and transported in warm (37 °C) physiological saline (0.9% NaCl) solution. Upon arrival, ovaries were washed three times with warm (37 °C) 0.9% NaCl, followed by prewarmed (37 °C) 70% ethanol washing. Granulosa cells were aspirated from small follicles (3-5 mm) in 15-mL tubes containing prewarmed (37 °C) phosphate-buffered saline, calcium- and magnesium-free (PBS-CMF) and then kept standing for 15 min at 37 °C to let the cellular debris settled down at the bottom. Subsequently, the supernatants containing the granulosa cells were collected and centrifuged at 750*g* for 7 min to get granulosa cell pellets. Afterwards, the cell viability and concentration were checked under the microscope using trypan blue staining. Ultimately, a total of 2.5 × 10^5^ cells per well was cultured in CytoOne® 24-well plate (Starlab GmbH, Hamburg, Germany) containing 600-µL DMEM/F-12 medium (Sigma-Aldrich Chemie GmbH, Taufkirchen, Germany) supplemented with 10% fetal bovine serum (FBS) (Gibco®, Karlsruhe, Germany) and 1% penicillin/streptomycin and incubated at 37 °C in 5% CO_2_.

## Small interference RNA transfection

Briefly, sub-confluent granulosa cells were transfected with 200 nM siRNA targeting *NRF2* (siRNA-*NRF2*) or scrambled siRNA as a negative control (siRNA-ve) (Exiqon, Vedbaek, Denmark) in the presence of Lipofectamine® 2000 (Invitrogen, Carlsbad, California, USA) as transfection reagent diluted in Opti-MEM I reduced-serum medium (Invitrogen, Carlsbad, California, USA). Afterwards, cells were incubated for 24 h in a humidified 5% CO_2_ incubator at 37 °C. Thereafter, cells were harvested and used for further analyses.

## Cell proliferation assay

A total of 1.5 × 10^4^ cells were cultured in 96-well plates using the same procedure described above. Cells from both experiments were used for proliferation assay using CCK-8 kits (Dojindo EU GmbH, München, Germany) after 24 h post-treatment either with H_2_O_2_ or siRNA according to the manufacturer’s protocol. The proliferation rate was indicated by the absorbance at 450 nm wavelength using Synergy™ H1 Multi-Mode Reader (BioTek Germany, Bad Friedrichshall, Germany).

## Intracellular ROS detection

Granulosa cells were cultured in a 96-well plate and then the sub-confluent cells were treated with H_2_O_2_. After 24 h, cells were co-incubated with FBS-free medium containing 75 µM 2′,7′-dichlorodihydrofluorescein diacetate (H2DCFDA) (Invitrogen, Carlsbad, California, USA) for 20 min at 37 °C in 5% CO_2_. Subsequently, the cells were washed twice with PBS-CMF, and then, the images were captured using a green-fluorescence filter of the inverted fluorescence microscope (Leica DM IRB, Leica, Wetzlar, Germany). The fluorescence intensity was quantified using ImageJ 1.48v software (National Institutes of Health, Maryland, USA, http://imagej.nih.gov).

## Mitochondrial activity assay

To assess the cellular mitochondrial activity, a total of 4 × 10^4^ live granulosa cells were seeded into 8-well slide chamber according to the manufacturer’s protocol. Briefly, the granulosa cells were co-incubated with 200 nM MitoTracker red dye (MitoTracker® Red CMXRos, M7512; Invitrogen) for 30 min in a CO_2_ incubator. Then, the cells were rinsed twice with PBS-CMF and subsequently fixed with 4% paraformaldehyde overnight at 4 °C. Fixed cells were mounted with a Vectashield mounting medium containing DAPI. Finally, the images were acquired using confocal microscopy CLSM LSM-780 and the fluorescent intensity was analyzed with ImageJ 1.48v software (National Institutes of Health, Maryland, USA, http://imagej.nih.gov).

## Lipid accumulation assay

Cells were cultured in a 96-well plate and treated with either H_2_O_2_ or siRNA. Twenty-four hours post-treatment, plates containing granulosa cells were gently rinsed with 50 µL of PBS-CMF. Subsequently, cells were fixed using 4% paraformaldehyde overnight at 4 °C and then subjected to lipid accumulation assay. Briefly, a working solution was prepared by mixing three parts (30 mL) of oil red O (Sigma-Aldrich Chemie GmbH, Munich, Germany) from stock solution with 2 parts (20 mL) deionized water and then stored at room temperature for 10 min followed by filtration via filter paper. After removing the 4% paraformaldehyde, each well was gently rinsed by sterile water followed by co-incubation with 50 µL of 60% isopropanol at room temperature for 5 min. Afterwards, the isopropanol was poured off and 100-µL working solution of oil red O was pipetted to each well and left for 5 min. Following this, cells were rinsed with tap water and the images were captured using an inverted microscope with 40× magnification.

## DNA fragmentation assay

The fragmented DNA was isolated according to the Abcam protocol (Cambridge, UK). Briefly, the floated and adhered cells were collected and then lysed in 500 µL of 10 mM Tris (pH 7.4), 5 mM EDTA, 0.2% Triton-X100 followed by vortexing for several times. The lysate was incubated on ice for 30 min followed by centrifugation at 27,000*g* for 30 min. Subsequently, the supernatants were divided into two parts and 50-µL ice-cold 5 M NaCl was added to each part coupled with several times vortexing. Subsequently, a mixture containing 600 µL ethanol and 150-µL 3 M sodium acetate (pH 5.2) was added to each aliquot with gentle mixing by pipetting and then incubated at − 80 °C for 1 h followed by centrifugation for 20 min at 20,000*g*. After discarding the supernatant, the pellets were pooled together and re-dissolved in 10 mM Tris and 5 mM EDTA accompanied by adding DNase-free RNase 10 mg/mL and then incubated in a water bath at 37 °C for 5 h. Afterwards, a combination of 25 µL proteinase K at 20 mg/mL (Qiagen GmbH, Hilden, Germany) and 40 µL buffer (100 mM Tris pH 8.0, 100 mM EDTA, and 250 mM NaCl) was added and subsequently incubated overnight at 65 °C. Thereafter, the DNA was extracted using phenol/chloroform/isoamyl alcohol mixture (25:24:1) (Biomol GmbH, Hamburg, Germany) and then precipitated using ethanol. The supernatant was carefully removed followed by air drying. The pellets were resuspended in 15-µL 1× TE buffer (Carl Roth GmbH, Karlsruhe, Germany). Finally, the samples were electrophoretically separated using a 2% agarose gel containing ethidium bromide and then visualized using ChemiDoc™ XRS+ system (Bio-Rad Laboratories GmbH, Germany).

## RNA isolation and cDNA synthesis

Total RNA from granulosa cells was extracted using the miRNeasy® mini kit (Qiagen GmbH, Hilden, Germany) following the manufacturer’s protocol. Before starting the total RNA extraction, granulosa cells were washed using 1× PBS to remove the remaining culture medium. At the end of the extraction protocol, trapped RNAs in the membrane of spin column were eluted using 30 μL RNase-free water. RNA concentration was assessed using NanoDrop 8000 UV-Vis Spectrophotometers (Thermo Scientific, Wilmington, USA). Equal amounts of total RNA were reverse transcribed using the RevertAid first-strand cDNA synthesis kit (Thermo Fisher Scientific, Schwerte, Germany). Briefly, a combination from oligo-dT and random primer was added to each sample followed by incubation at 65 °C for 5 min. After incubation time, a mixture containing 1 µL of RiboLock RNase inhibitor, 2 µL of 10 mM dNTP mix, 4 µL of 5× reaction buffer, and 2 µL of MMuLV reverse transcripts was added to each sample. Afterwards, samples were incubated at 25 °C for 5 min, then 37 °C for 60 min followed by 70 °C for 5 min to terminate the reaction. Ultimately, the cDNA was kept at − 20 °C for further analysis.

## Real-time quantitative PCR

The expression levels of candidate TFs (*NRF2*, *NOTCH1*, *SREBF1*, *SREBF2*, *KLF4*, *KLF9*, *FOS*, *FOSB*, *IRF7*, *E2F1*, and *E2F4*), the *KLF4* downstream target genes (*CCNB1*, *CCND2*, and *BAX*), endoplasmic reticulum stress marker (*Grp78*, *Grp94*, and *Calpain1*), cell differentiation markers (*CYP11A1*, *CYP19A1*, *STAR*, *INHBa*, *EGFR*, and *FOXO1*), and apoptosis-related genes (*BCL2L1* and *CASP3*) were quantified using iTaq™ Universal SYBR® Green Supermix (Bio-Rad Laboratories GmbH, München, Germany) in Applied Biosystem® StepOnePlus™ (Applied biosystems, Foster City, CA, USA). The primers were designed using the primer3 online tool (http://bioinfo.ut.ee/primer3-0.4.0/) (Supplementary Table 1). Subsequently, the specificity of the primers was confirmed by sequencing using the GenomeLab™ GeXP Genetic Analysis System (Beckman Coulter GmbH, Krefeld, Germany). The mRNA expression data were analyzed using comparative Ct (2^−ΔΔCt^) methods (Livak and Schmittgen 2001) and the geometric mean of the expression level of *ACTB* and *GAPDH* was used for normalization after checking the expression stability with NormFinder software (Andersen et al. 2004).

## Immunoblotting

Total protein from granulosa cells was isolated using 1× passive lysis buffer (Promega GmbH, Mannheim, Germany) and the concentration was determined using Coomassie Protein Assay Reagent (Life Technologies GmbH, Darmstadt, Germany). Thirty-five micrograms of protein was loaded onto 12% SDS-polyacrylamide gel. After electrophoresis, proteins were transferred onto a nitrocellulose membrane (Bio-Rad Laboratories GmbH, Germany) using Trans-Blot® SD Semi-Dry Transfer Cell (Bio-Rad Laboratories GmbH, Germany). Thereafter, the membrane was blocked in Roti®-Block (Carl Roth GmbH, Germany) for 1 h at room temperature. The membrane was incubated overnight at 4 °C with polyclonal primary antibodies specific for KLF4 (1:100), Grp78 (1:300), PRKAA 1/2 (1:350), StAR (1:250), and ACTB (1:500) mouse monoclonal antibody (Santa Cruz Biotechnology Inc, Germany). After the incubation with the primary antibody, the membrane was incubated with goat anti-rabbit or goat anti-mouse (1:5000; Santa Cruz Biotechnology Inc, Germany) secondary antibody with the respective candidate primary antibody for 1 h at room temperature. The proteins were visualized using Clarity™ Western ECL Substrate (Bio-Rad Laboratories Inc, USA). The images were captured using the ChemiDoc™ XRS+ system (Bio-Rad Laboratories GmbH, Germany). Subsequently, the membrane was stripped using a middle stripping buffer in order to remove primary and secondary antibodies followed by blocking and incubation with another antibody using the same protocol. As shown in supplementary Fig. 3, in both control and H_2_O_2_-treated cells, the StAR protein was detected at 30 kDa.

## Immunocytochemistry

Localization and subsequent quantification of NRF2 and KLF4 proteins were conducted using the immunocytochemistry technique. For this, granulosa cells were seeded into 8-well slide chamber and subsequently treated with either H_2_O_2_ or siRNA. Twenty-four hours post-treatment, granulosa cells were fixed using 4% paraformaldehyde and kept at 4 °C overnight. Subsequently, the cells were rinsed three times with PBS-CMF and then incubated with 0.3% Triton X-100 (Roche Diagnostics GmbH, Mannheim, Germany) for 10 min followed by blocking with 3% donkey serum (Sigma-Aldrich Chemie GmbH, Munich, Germany) for 1 h at room temperature. Then, the cells were co-incubated with the primary antibody for NRF2 (1:100) or KLF4 (1:100) at 4 °C overnight. Thereafter, the cells were washed three times with PBS-CMF followed by co-incubation with fluorescent secondary antibody (Lifespan Biosciences, Alexa Fluor goat anti-rabbit 1:350) for 3 h at 37 °C in dark. Thereafter, the cells were washed three times with PBS-CMF and subsequently mounted with Vectashield mounting medium containing DAPI. Finally, the cells were visualized under confocal microscopy CLSM LSM-780 and the fluorescence intensity of proteins was analyzed using ImageJ 1.48v software (National Institutes of Health, Maryland, USA, http://imagej.nih.gov).

## Statistical analysis

Data were represented as means ± SEM from at least three independent replicates with different pools of cells. Data were statistically analyzed with Student’s two-tailed *t* test using GraphPad Prism software version 7 (GraphPad Software, La Jolla, California, USA). The difference between means was considered significant when *P *value ≤ *0.05*.

## Results

### Oxidative stress impaired granulosa cell functions

**Fig. 1:**
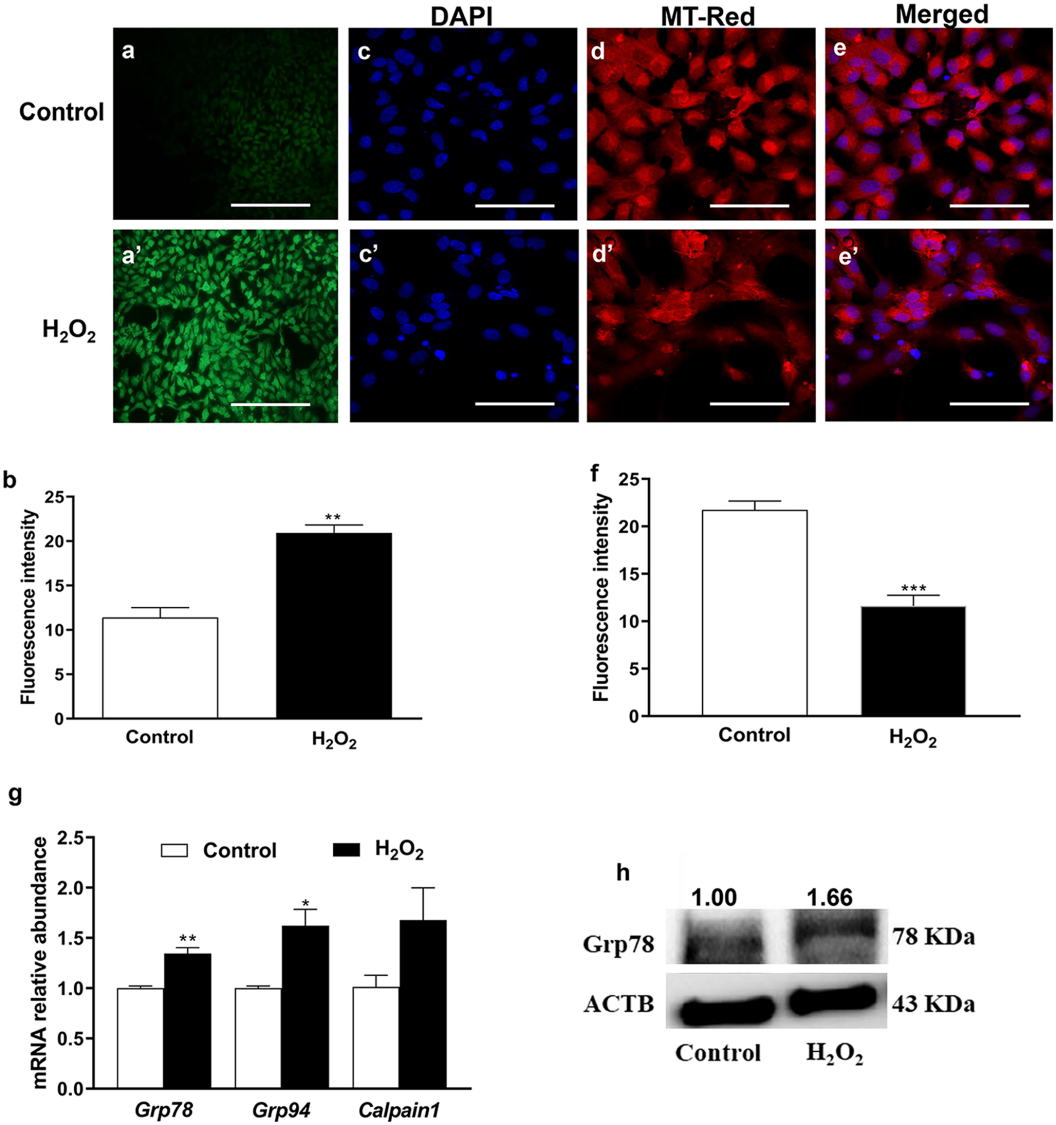
Intracellular reactive oxygen species (ROS) accumulation **a**, **b**, mitochondrial activity **c-f**, the relative mRNA expression profile of endoplasmic reticulum stress marker genes **g**, and protein level of Grp78 **f** of bovine granulosa cells under oxidative stress conditions. Green color **a**, **a’** indicates the fluorescence of the 2′,7′-dichlorodihydrofluorescein diacetate (H2DCFDA). The blue color **c**, **c’** indicates the nuclear staining using 40,6-diamidino-2-phenylindole (DAPI) and the red color **d**, **d’** indicates mitochondrial activity. The fluorescence and band intensity were measured by ImageJ software. Data were statistically analyzed with Student’s two-tailed *t* test and are shown as means ± SEM of three independent replicates with different pools of cells (*p ≤ *0.05*, **p ≤ *0.01*, ***p ≤ *0.001*). Scale bars: 100 µm **a**, **a’**, 20 µm **c-e’**

Bovine granulosa cells treated with H_2_O_2_ exhibited shrinkage in shape and decreased confluency compared with untreated counterparts (Suppl. Figure 1). Moreover, exposure of cells to H_2_O_2_ elevated (*P* ≤ *0.01*) the intracellular ROS amount and decreased (*P* ≤ *0.001*) mitochondrial activity compared with the untreated controls (Fig. 1a-f). Moreover, the higher intracellular ROS accumulation in H_2_O_2_-challenged cells was accompanied by the induction of DNA fragmentation (Suppl. Figure 2). Overall, these results indicated that bovine granulosa cells treated with H_2_O_2_ resulted in the induction of oxidative stress phenotypes.

**Fig. 2:**
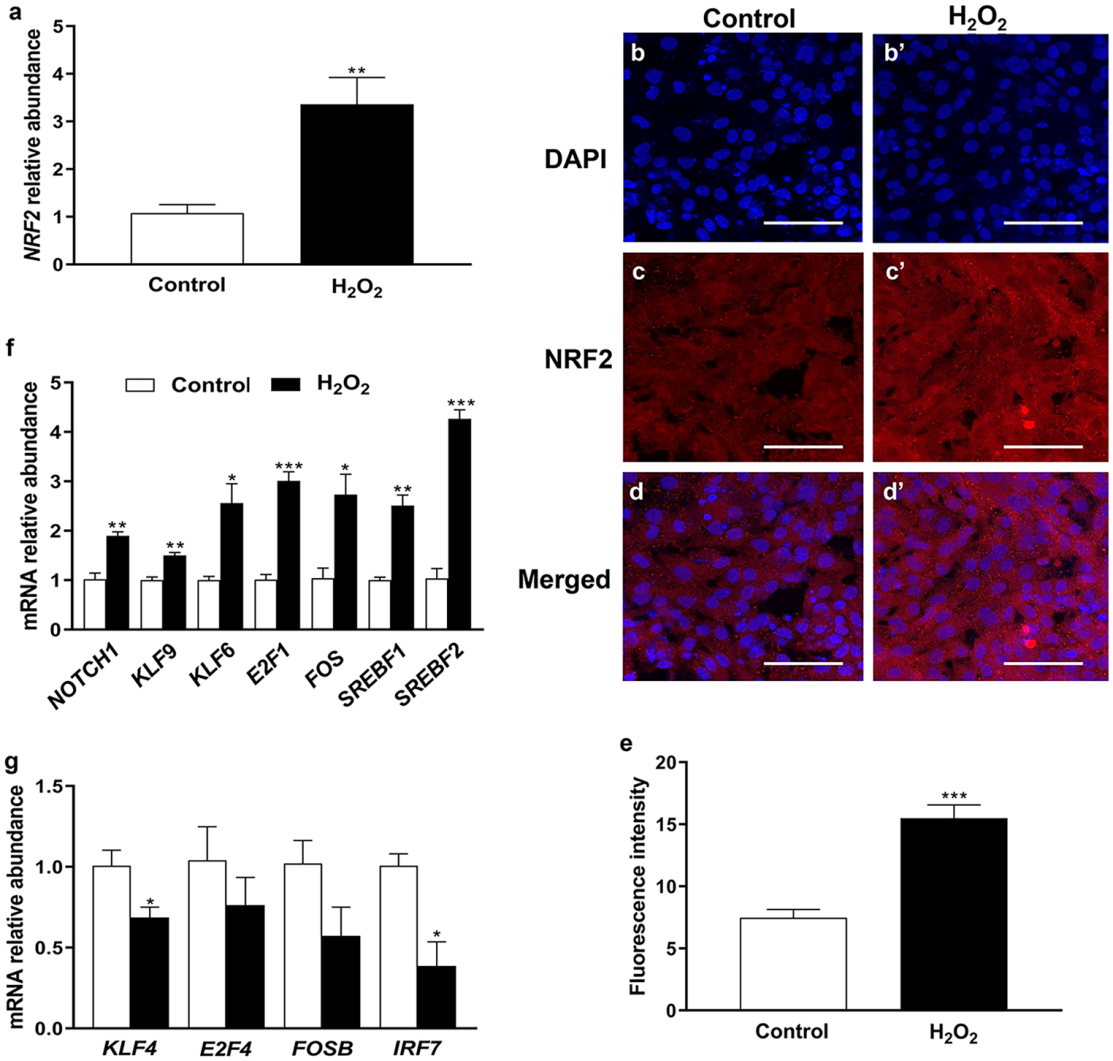
Relative abundance of the transcription factors in bovine granulosa cells exposed to oxidative stress. The expression pattern of *NRF2* mRNA **a** and its protein **b-e** in response to oxidative stress, whereby the blue color **b**, **b’** indicates the nuclear staining using 40,6-diamidino-2-phenylindole (DAPI) and the red color **c**, **c’ **indicates the NRF2 protein; the effect of oxidative stress on candidate transcription factors, which showed up **f** and downregulation **g**. The transcript expression data were normalized to internal *ACTB* and *GAPDH* as well as relatively compared with the control group. Data were statistically analyzed using Student’s two-tailed *t* test and are represented as means ± SEM of triplicates (*p ≤ *0.05*, **p ≤ *0.01*, ***p ≤ *0.001*). The ImageJ software was utilized to calculate the fluorescence intensity of NRF2 protein. Scale bars: 20 µm **b-d’**

## H_2_O_2_-induced oxidative stress coupled with endoplasmic reticulum stress

The mRNA expression level of glucose-regulated protein (*Grp*)*78* and *Grp94* was significantly (*P* ≤ *0.01* and *P* ≤ *0.05*, respectively) higher in H_2_O_2_-treated cells compared with the controls (Fig. 1g). Furthermore, the protein level of Grp78 was in agreement with the corresponding mRNA expression level (Fig. 1h). However, the expression level of *Calpain1* did not show any significant differences.

## H_2_O_2_-induced oxidative stress altered the expression pattern of candidate TFs

The temporal expression level of the candidate TFs which have pivotal roles in stress response (*NRF2*, *FOS*, and *FOSB*), apoptosis, differentiation, and proliferation (*KLF4*, *KLF6*, *KLF9*, and *NOTCH1*), cell cycle (*E2F1* and *E2F4*), fatty acids and cholesterol biosynthesis (*SREBF1* and *SREBF2*), and immune response (*IRF7*) was investigated. Upon oxidative stress induction, the expression pattern of *NRF2* was significantly increased (*P* ≤ *0.01*, Fig. 2a). Similarly, the mRNA transcript level of *NOTCH1*, *FOS*, *SREBF1*, *SREBF2*, *KLF6*, *KLF9*, and *E2F1* was significantly higher (*P* ≤ *0.05*) in the H_2_O_2_-treated group compared with the untreated controls (Fig. 2f). Contrary to this, the expression pattern of *KLF4* and *IRF7* was significantly lower (*P* ≤ *0.05*) in H_2_O_2_-treated granulosa cells compared with the untreated control group (Fig. 2g). However, no significant differences were observed in the expression of *E2F4* and *FOSB* (Fig. 2g).

## Oxidative stress influenced the protein level of NRF2 and KLF4

**Fig. 3:**
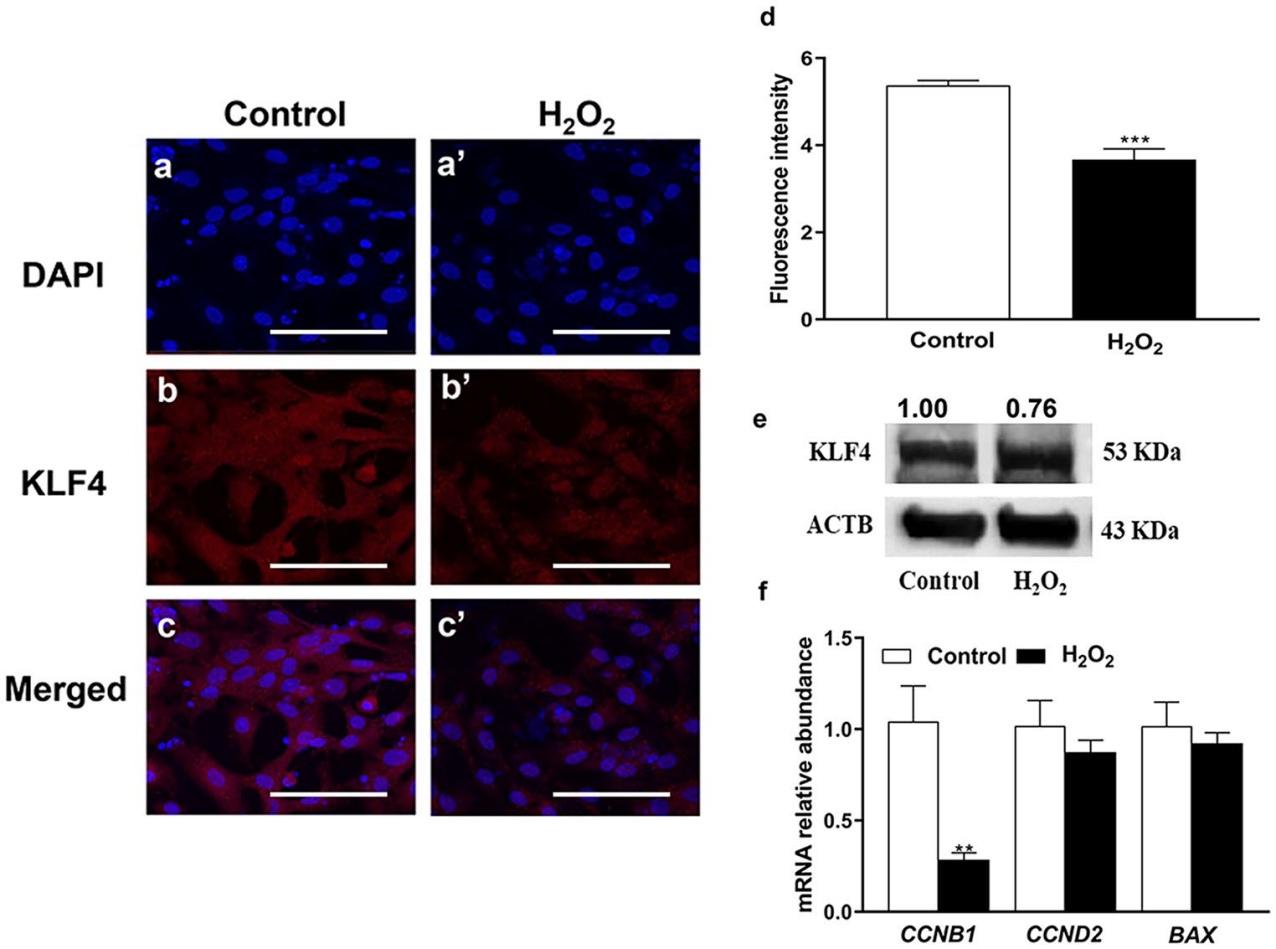
Impact of oxidative stress on the protein level of KLF4 **a-e** and its target genes **b**. The blue color **a**, **a’** in immunocytochemistry indicates the nuclear staining using 40,6-diamidino-2-phenylindole (DAPI) and the red color **b**, **b’** indicates KLF4 protein. The mRNA data were normalized to *ACTB* and *GAPDH* as well as relatively compared with the control group. Data represent means ± SEM from triplicates (**p ≤ *0.01*, ***p ≤ *0.001*). The Western blot protein analysis of KLF4 **e** was compared with the internal expression of ACTB. The ImageJ software was utilized to calculate the fluorescence intensity of KLF4 protein. Scale bars: 20 µm **a-c’**

The protein level of NRF2 and KLF4 was assessed using immunocytochemistry assay after exposure of granulosa cells to oxidative stress. Similar to mRNA expression, the protein abundance of NRF2 was significantly upregulated (*P* ≤ *0.001*) in H_2_O_2_-challenged cells compared with controls (Fig. 2c, c’, and e). Furthermore, the NRF2 protein was found to be localized in the nucleus of treated cells. However, the protein abundance of KLF4 was significantly lower (*P* ≤ *0.001*) in the H_2_O_2_-challenged cells (Fig. 3a-e).

## Alterations of downstream genes of KLF4 under oxidative stress condition

The downregulation of *KLF4* in the H_2_O_2_-treated group was accompanied by a significant reduction (*P* ≤ *0.01*) of *CCNB1* (Fig. 3f). However, the mRNA expression of *CCND2* and *BAX* was not significantly different between H_2_O_2_-treated and untreated groups (Fig. 3f).

## Oxidative stress induced lipid accumulation

**Fig. 4:**
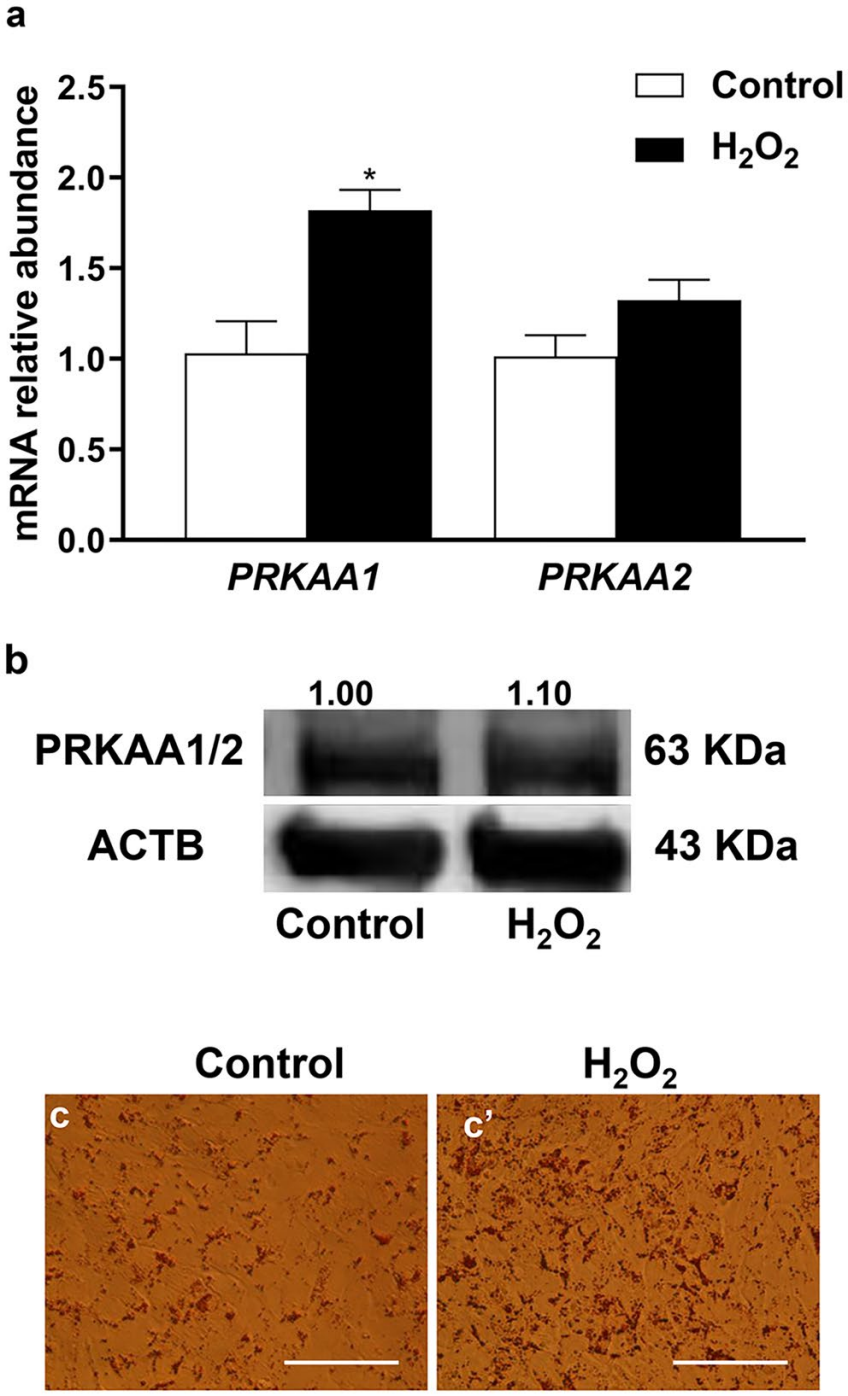
The effect of oxidative stress on mRNA expression of *PRKAA1* and *PRKAA2* **a**, the protein level of PRKAA1/2 **b**, and lipid accumulation **c**, **c’** in bovine granulosa cells. The red color **c**, **c’** indicates the oil red O stain. Data were statistically analyzed using Student’s two-tailed *t* test and represented as means ± SEM of triplicates (*p ≤ *0.05*). Scale bars: 25 µm **c**, **c’**

In addition to KLF4, TFs involved in fatty acids and cholesterol biosynthesis (*SREBF1* and *SREBF2*) and cellular lipid accumulation were investigated under oxidative stress condition. Results showed a significant increment (*P* ≤ *0.05*) in mRNA expression of *PRKAA1* (Fig. 4a) as an upstream gene of *SREBF1* and *SREBF2*. Additionally, the PRKAA1/2 protein level (Fig. 4b) and lipid accumulation (Fig. 4c and c’) were higher in the H_2_O_2_-challenged cells compared with control ones.

## Oxidative stress-induced granulosa cell differentiation marker genes

**Fig. 5:**
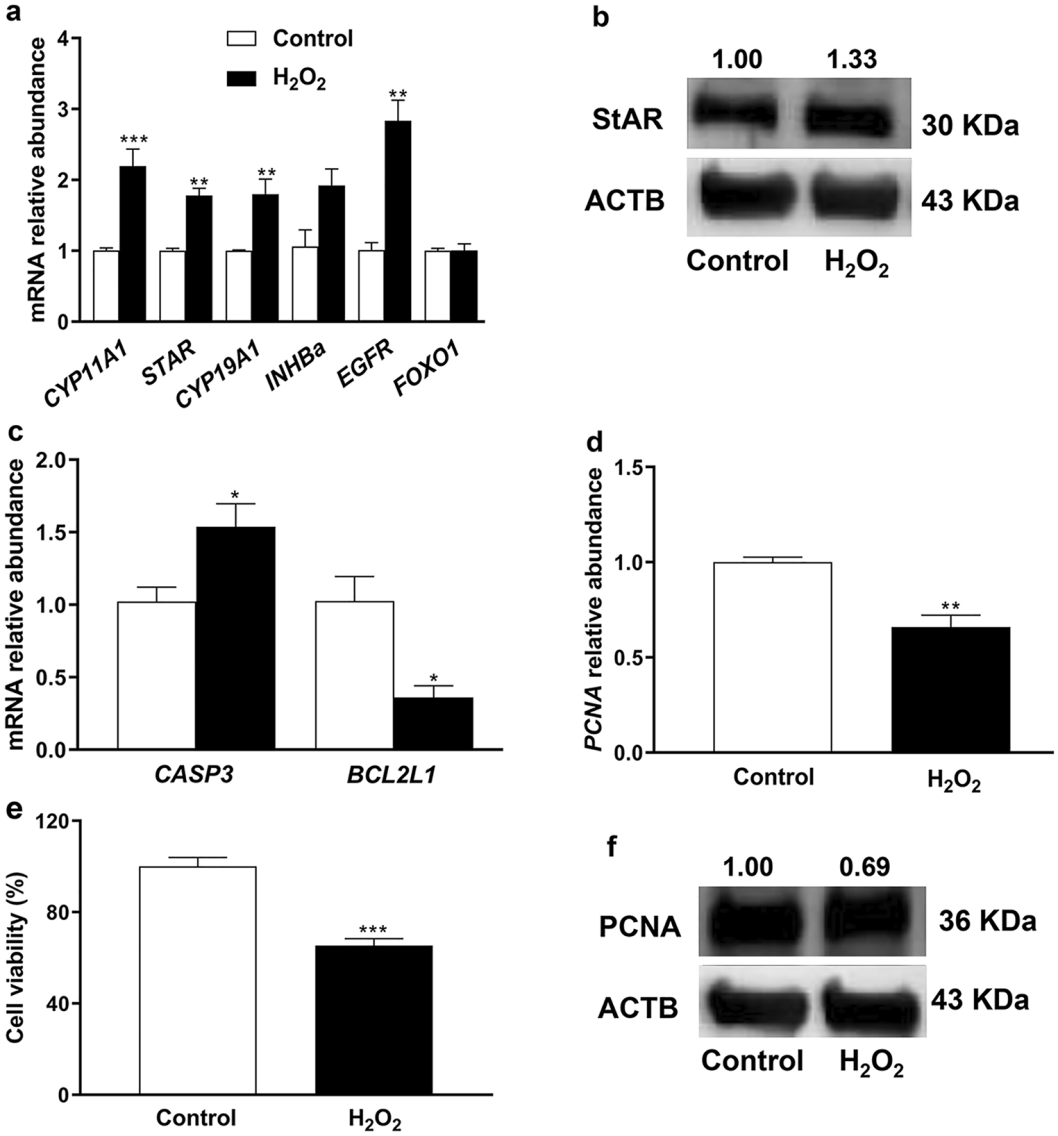
The mRNA expression pattern of differentiation-related genes (**a**), StAR protein (**b**), expression pattern of anti-apoptotic, pro-apoptotic **c**, proliferation marker **d **genes, cell viability **e**, and PCNA protein level **f** in bovine granulosa cells exposed to H_2_O_2_-induced oxidative stress. The geometric mean of *ACTB* and *GAPDH* was used for mRNA normalization and the data were relatively compared with the control group. The band intensity of immunoblotting **f** was measured by ImageJ software. Data were statistically analyzed with Student’s two-tailed *t* test and are represented as means ± SEM of triplicates (*p ≤ *0.05*, **p ≤ *0.01*, ***p ≤ *0.001*). The level of ACTB protein was used as an internal control in protein analysis

To clarify whether the exposure of bovine granulosa cells to oxidative stress affected their ability to differentiate, the expression pattern of differentiation marker genes was investigated. The mRNA expression of *CYP11A1*, *CYP19A1*, and *STAR* was significantly upregulated (*P* ≤ *0.001*,* P* ≤ *0.01*, and *P* ≤ *0.01*, respectively) upon exposure of granulosa cells to H_2_O_2_ (Fig. 5a). Additionally, the protein abundance of StAR showed upregulation in challenged cells (Fig. 5b). Moreover, the expression pattern of *EGFR* was significantly upregulated (*P* ≤ *0.01*) in the H_2_O_2_-treated group compared with untreated controls. However, no significant differences were noticed in the mRNA expression level of Inhibin Beta A (*INHBa*) and Forkhead box O1 (*FOXO1*) between the treatment groups (Fig. 5a).

## Oxidative stress induced the expression of the pro-apoptotic gene and subsequently impaired granulosa cell proliferation

Unlike *BAX*, the mRNA level of *CASP3*, a pro-apoptotic gene, was significantly increased (*P* ≤ *0.05*) in H_2_O_2_-challenged cells (Fig. 5c). However, the expression of the anti-apoptotic marker gene (*BCL2L1*) was significantly decreased (*P* ≤ *0.05*) under the H_2_O_2_ challenge. On the other hand, the mRNA and protein expression level of the proliferation marker gene (*PCNA*) was significantly decreased (*P* ≤ *0.01*) under oxidative stress condition (Fig. 5 d and f). Accordingly, a reduction (*P* ≤ *0.001*) in cell proliferation rate was observed upon exposure of granulosa cells to H_2_O_2_ challenge (Fig. 5e).

## Selective knockdown of *NRF2* altered the expression pattern of stress-related candidate TFs

**Fig. 6:**
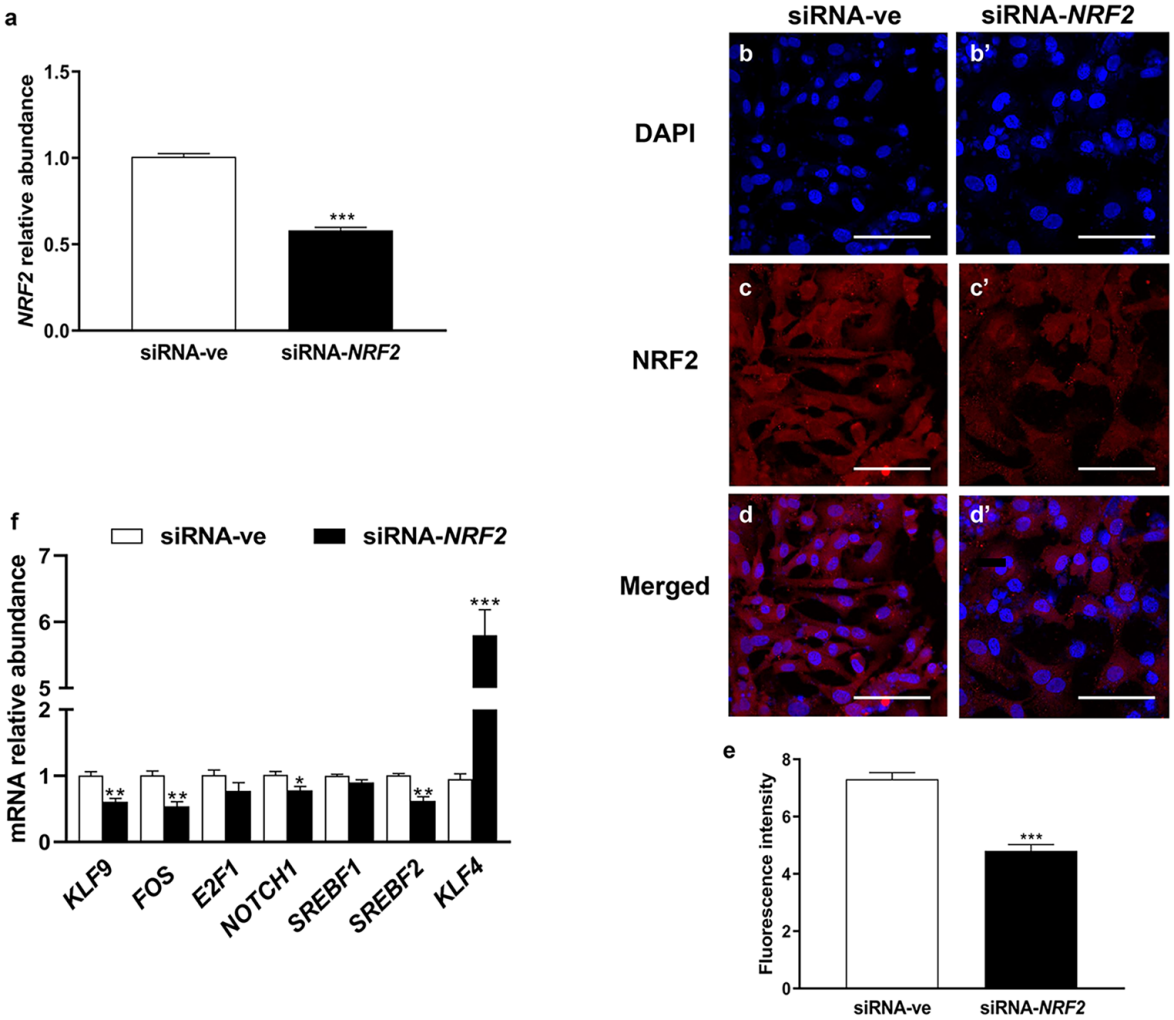
Selective knockdown of *NRF2* in cultured bovine granulosa cells altered the expression of candidate transcription factors; the efficiency of utilizing small interference (siRNA) on the mRNA **a** and protein **b–e** levels of *NRF2*; the impact of siRNA-mediated *NRF2* knockdown on the expression pattern of candidate transcription factors **f**. The mRNA data were normalized to internal control of *ACTB* and *GAPDH* as well as the data were relatively compared with the negative control group (siRNA-ve). The blue color **b**, **b’** indicates the nuclear staining using 40,6-diamidino-2-phenylindole (DAPI) and the red color in immunocytochemistry **c**, **c’ **indicates NRF2 protein. Data were analyzed with Student’s two-tailed *t* test and are represented as means ± SEM of triplicates (*p ≤ *0.05*, **p ≤ *0.01*, ***p ≤ *0.001*). The ImageJ software was used to measure the fluorescence intensity of NRF2 protein. Scale bars: 20 µm **b–d’**

The granulosa cells transfected with siRNA targeting *NRF2* exhibited reduced expression of *NRF2* (*P* ≤ *0.001*; Fig. 6). Upon *NRF2* knockdown, the mRNA expression of *KLF9*, *FOS*, *NOTCH1*, and *SREBF2* was significantly downregulated (*P* ≤ *0.05*; Fig. 6f). Interestingly, the relative mRNA expression of *KLF4* was significantly increased (*P* ≤ *0.001*) by approximately fivefold. However, the mRNA expression levels of *E2F1* and *SREBF1* were not significantly altered.

## The consequence of *NRF2* knockdown on the expression of *KLF4* and its downstream genes and cell proliferation

**Fig. 7:**
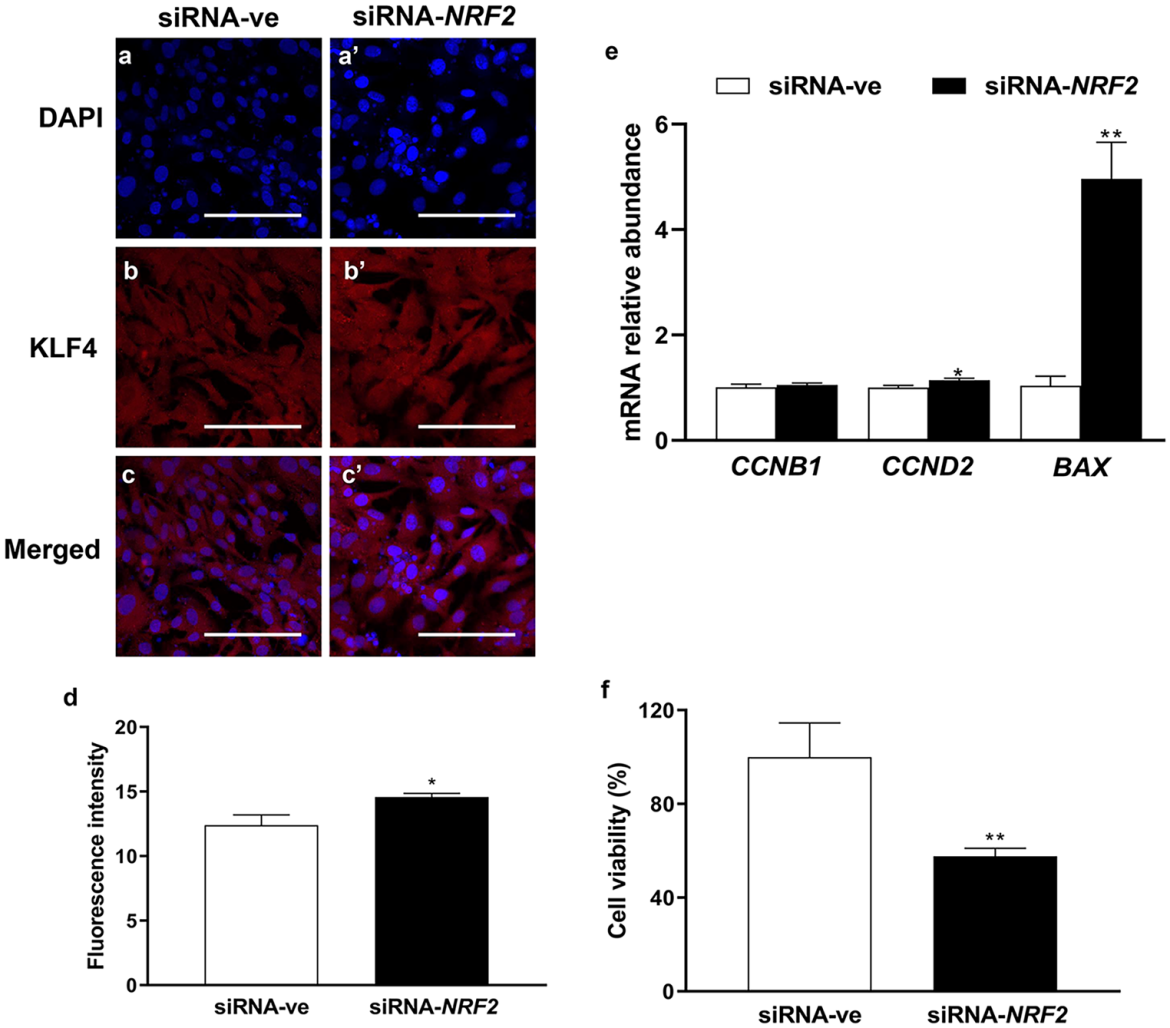
The effect of transfection with small interference RNA targeting *NRF2* (siRNA-NRF2) on the protein level of KLF4 **a-d** and its target genes **e** as well as cell proliferation rate **f**. The blue color **a**, **a’** indicates the nuclear staining using 40,6-diamidino-2-phenylindole (DAPI) and the red color **b**, **b’** indicates the KLF4 protein. Data were analyzed with Student’s two-tailed *t* test and are represented as means ± SEM of triplicates (*p ≤ *0.05*, **p ≤ *0.01*). The ImageJ software was used to measure the fluorescence intensity of KLF4 protein. Scale bars: 20 µm **a-c’**

Targeted suppression of *NRF2* resulted in a significant increase in the protein level of KLF4 (Fig. 7a-d). Next, to demonstrate the effect of *NRF2* knockdown and induced expression of KLF4 on granulosa cell functions, the expression pattern of KLF4 downstream genes and cell proliferation markers was investigated. Consequently, the mRNA expression of *CCND2* was upregulated (*P* ≤ *0.05*; Fig. 7e). Intriguingly, the expression pattern of *BAX* was upregulated (*P* ≤ *0.01*) accompanied by a significant reduction (*P* ≤ *0.01*) in cell proliferation rate (Fig. 7f).

## *NRF2* knockdown altered the expression pattern of genes associated with differentiation and lipid accumulation

**Fig. 8:**
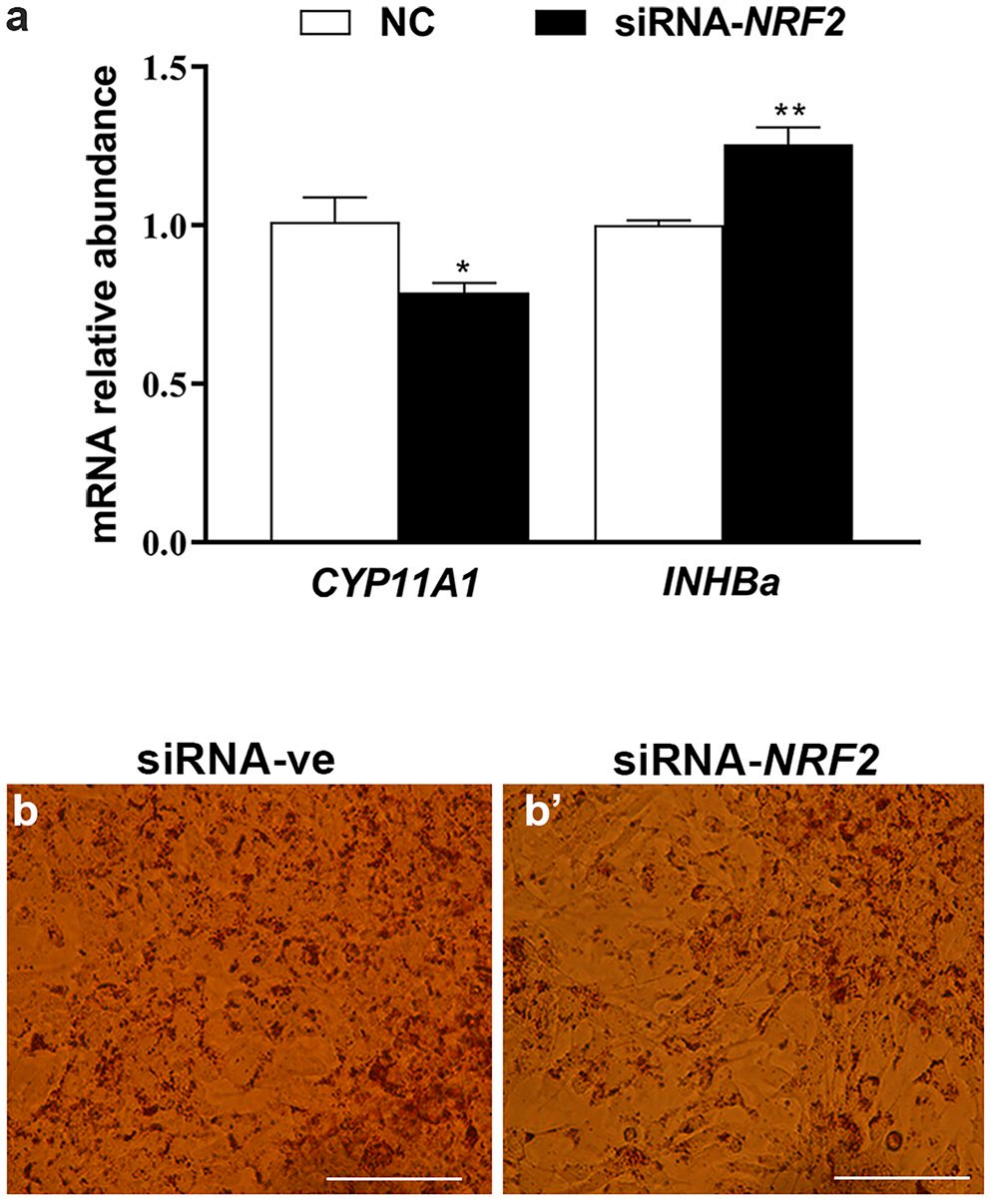
The effect of siRNA-mediated *NRF2* knockdown **a** and lipid accumulation **b**, **b’**. The red color **b**, **b’** indicates the oil red O stain. The expression level of *ACTB* and *GAPDH* was used to normalize mRNA transcript and the data were relatively compared with the negative control group (siRNA-ve). Data were analyzed with Student’s two-tailed *t* test and represented as means ± SEM of triplicates (*p ≤ *0.05*, **p ≤ *0.01*). Scale bars: 25 µm **b**, **b’**

The mRNA expression level of the *CYP11A1* transcript was significantly reduced (*P* ≤ *0.05*) in siRNA-*NRF2*-transfected cells (Fig. 8a) and the mRNA level of *INHBa* was significantly increased (*P* ≤ *0.01*). On the other hand, the *NRF2* knockdown resulted in dysregulation of fatty acids and cholesterol biosynthesis TFs, which was confirmed by lower lipid accumulation (Fig. 8b and b’).

## Discussion

In the current study, granulosa cells were exposed to moderate oxidative stress via 5 µM H_2_O_2_ treatment as we have used previously (Saeed-Zidane et al. 2017). The H_2_O_2_, one of the non-radical groups of ROS, is diffused through the cell membrane (Tamma et al. 2018) and subsequently increased the accumulation of intracellular ROS level (Sohel et al. 2019; Zhang et al. 2016). This strategy is orchestrated during the ovulation process by leukocytes, which are deemed to be the source of ROS (Sugino 2005).

Upon increasing intracellular ROS accumulation, the free radicals attack the cellular bioactive molecules such as DNA, lipids, and proteins (Guo et al. 2013; Kadenbach et al. 2004; Zhang et al. 2016), as has been found in the current investigation (Supp. Figure 2). Moreover, the compromising of mitochondrial DNA could cause mutations and subsequently mitochondrial dysfunction (Hollensworth et al. 2000). The permeability of the mitochondrial membrane is prone to lipid peroxidation resulted from the higher levels of ROS, which ultimately impairs the functions of mitochondria and decreases its activity (Guo et al. 2013; Kirkinezos et al. 2005), as shown in the present study (Fig. 1d, d’, and f). This could lead to disturbing the cellular metabolism (Kadenbach et al. 2004), increase apoptosis (Yang et al. 1997), and decrease cell proliferation as well as alter the cell cycle (Antico Arciuch et al. 2012; Saeed-Zidane et al. 2017). Besides that, H_2_O_2_-induced oxidative stress contributes to altering the protein folding mechanism and induction of endoplasmic reticulum stress (Kim et al. 2016; Malhotra et al. 2008). The endoplasmic reticulum stress has a dual role in ovarian functions either during atresia (Lin et al. 2012) or maturation of the follicle and corpus luteum formation (Huang et al. 2017), which was confirmed in our study by the upregulation of endoplasmic reticulum marker genes (*Grp78* and *Grp94*) mRNA and the protein level of Grp78 (Fig. 1g and h). Both *Grp78* and *Grp94* contribute to the maintenance of endoplasmic reticulum homeostasis and induction of apoptosis via activation of caspase under endoplasmic reticulum stress condition (Liu et al. 2011).

Besides their roles in defense mechanisms in a cell-dependent manner, several TFs have been proven to play a pivotal role in controlling ovarian functions (Sirotkin 2010). For instance, highly abundant NRF2 protein was found to be localized in the porcine follicular cells of the preovulatory follicle and newly formed corpus luteum (Likszo et al. 2019) to trigger the release of antioxidant machinery and subsequently protect the cells from apoptosis (Khadrawy et al. 2019; Likszo et al. 2019; Saeed-Zidane et al. 2017; Sohel et al. 2019). Similarly, we found upregulation of NRF2 expression in granulosa cells exposed to oxidative stress (Fig. 2), which is the master of TFs involved in oxidative stress response. Furthermore, KLF9-induced apoptosis has been found to be regulated by oxidative stress in cancer cells via NRF2 (Zucker et al. 2014), as has been found in the current study (Fig. 2). Therefore, we speculated the potential role of NRF2 in the regulation of other TFs under oxidative stress conditions and subsequently alteration of ovarian functions.

In ovarian cells, the expression of *KLF4* and *KLF13* mRNA is regulated by luteinizing hormone (LH) or/and insulin-like growth factor 1 (*IGF1*) in follicular granulosa cells (Natesampillai et al. 2008), while the dysregulation of *KLF2* and *KLF4* expression was noticed in polycystic ovary syndrome (PCOS) compared with the normal ovaries (Jansen et al. 2004). In the present study, the mRNA expression of *KLF4* was significantly decreased under oxidative stress conditions (Fig. 2g), which was subsequently confirmed by the reduction of its protein level (Fig. 3b, b’, and d) and its *CCND2* target gene (Fig. 3f). *CCND2*, which is known to be involved in the regulation of apoptosis (Li et al. 2015) and cell cycle (Choi and Roh 2019), is found to be associated with the reduction in cell proliferation rate (Fig. 5e). Furthermore, our previous study showed a reduction in the expression of *CCND2* and subsequently cell cycle arrest at the G_2_/M phase in H_2_O_2_-treated granulosa cells (Saeed-Zidane et al. 2017). The upregulation of KLF4 was found to interfere with the expression of CYP19A1 (Choi et al. 2019), which is responsible for estrogen biosynthesis. However, the reciprocal expression pattern of KLF4 and NRF2 was found during *NRF2*-knockdown (Fig. 6) resulting in the induction of *BAX* (Fig. 7e), which is responsible for stimulating the apoptosis in granulosa cells (Yang et al. 2017). This indicates the novel protective role of the NRF2 signaling pathway by inhibiting KLF4-induced apoptosis (Li et al. 2015) under oxidative stress conditions.

The reduction of *KLF4* expression could be indirectly regulated by the higher expression of *SREBF2*, as has been shown in endothelial cells exposed to oxidative stress (Chen et al. 2015). *SREBF1* and *SREBF2* are TFs involved in the regulation of fatty acids and cholesterol biosynthesis (Shimano 2001). In the preovulatory follicle, the reduction of cholesterol as a consequence of LH-induced steroidogenesis leads to the stimulation of genes coding for cholesterol biosynthesis (Likszo et al. 2019). In agreement with that, our results showed that *SREBF1* and *SREBF2* were found to be upregulated in granulosa cells exposed to oxidative stress conditions (Fig. 2f), which was coupled with a reduction of *KLF4* expression and induction of genes responsible for steroidogenic enzymes (Fig. 5). On the other hand, the elevated ROS-induced endoplasmic reticulum stress (Fig. 1) was found to be responsible for increasing the SREBF2-cleavage (Colgan et al. 2007), which is in agreement with our results. Accordingly, the induction of those TFs could lead to increasing lipid accumulation and subsequently promoting of granulosa cell apoptosis and differentiation (Regan et al. 2018), as evidenced by the higher expression of differentiation marker genes (Fig. 5a) and apoptosis (Fig. 5c), as has been previously reported in granulosa cells treated with FSH (Liu et al. 2009). These facts indicate the significant contribution of ROS in granulosa cell transdifferentiation (Shkolnik et al. 2011). Additionally, the dysregulation of *SREBFs*’ levels resulted in reducing lipid accumulation upon *NRF2* knockdown, which indicates the potential role of NRF2 signaling in cellular metabolism through regulation of *SREBFs*’ family (Kamisako et al. 2014). This led to a reduction of *CYP11A1* as a differentiation marker gene in granulosa cells (Fig. 8a), which demonstrated the vital role of *NRF2* in harmonic with other TFs in the regulation of granulosa cell differentiation.

Moreover, the *E2F1*, one of E2Fs family involved in cell cycle regulation and apoptosis (Crosby and Almasan 2004), was found to be increased in H_2_O_2_-treated granulosa cells (Fig. 2f). Consistently, the upregulation of *E2F1* was accompanied by increasing the apoptosis in various cell types (Ginsberg 2002), which is evidenced by increasing DNA fragmentation (Suppl. Figure 2) and decreasing of cell proliferation rate (Fig. 5e) in the present study. Interestingly, as has been evidenced in the current study, there are E2F-binding sites in the NOTCH promotor resulting in the upregulation of NOTCHs’ family expression (Viatour et al. 2011).

There is clear evidence that the *NOTCH* signaling pathway is implicated in ovarian follicular development, through the spatial and temporal regulation of its associated genes, during follicular growth and selection (Murta et al. 2015; Vanorny et al. 2014; Zhang et al. 2016) as well as corpus luteum steroidogenesis and regression (Murta et al. 2015). Additionally, the stimulation of *NOTCH* signaling acts as a suppressor of apoptosis signal-regulating kinase 1 (*ASK1*) 1 and consequently p38 MAPK signaling pathway (Mo et al. 2013). Accordingly, the upregulation of the *NOTCH1* transcript in the H_2_O_2_-treated group (Fig. 2f) could be a defense mechanism to suppress the elevation of apoptosis under oxidative stress conditions. This is in agreement with the fact that the NOTCH signaling pathway is involved in the regulation of granulosa cell functions during follicle development (Jing et al. 2017). This novel results in bovine granulosa cells open a new window for further investigations related to TF cross-talk during oxidative stress and their association with apoptosis and differentiation.

Collectively, granulosa cells exposed to oxidative stress showed higher intracellular ROS accumulation accompanied by increased DNA fragmentation and reduced mitochondrial activity. The challenged cells activated the *NRF2* signaling pathway in parallel with other TFs involved in apoptosis and differentiation (Suppl. Figure 4), which was remarkably evidenced by the reduction of cell proliferation and induction of steroidogenic genes and lipid accumulation. Those impacts were reversed by *NRF2* knockdown. Therefore, the *NRF2* signaling pathway in cooperation with other stress-related TFs plays a vital role in the oxidative stress response through regulation of bovine granulosa cells apoptosis and differentiation, which showed its implication for follicular development.

## Supplementary Information

Below is the link to the electronic supplementary material.

Supplementary file1 (TIF 2321 KB)

Supplementary file2 (TIF 3598 KB)

Supplementary file3 (TIF 2068 KB)

Supplementary file4 (TIF 1111 KB)
