# Supplementary figures and images for: NRF2-mediated signaling is a master regulator of transcription factors in bovine granulosa cells under oxidative stress condition
Source: Cell Tissue Res. 2021 May 19;385(3):769–83. doi: 10.1007/s00441-021-03445-4 (PMC8526460; doi:10.1007/s00441-021-03445-4)

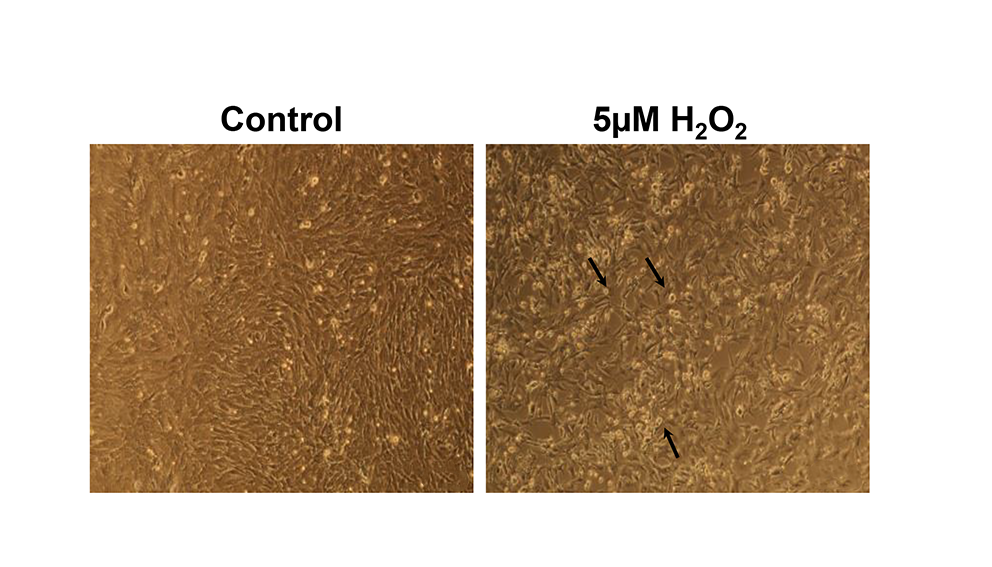

Supplement: Supplementary file 1 — Supplementary file1 (TIF 2321 KB) [file 441_2021_3445_MOESM1_ESM.tif]

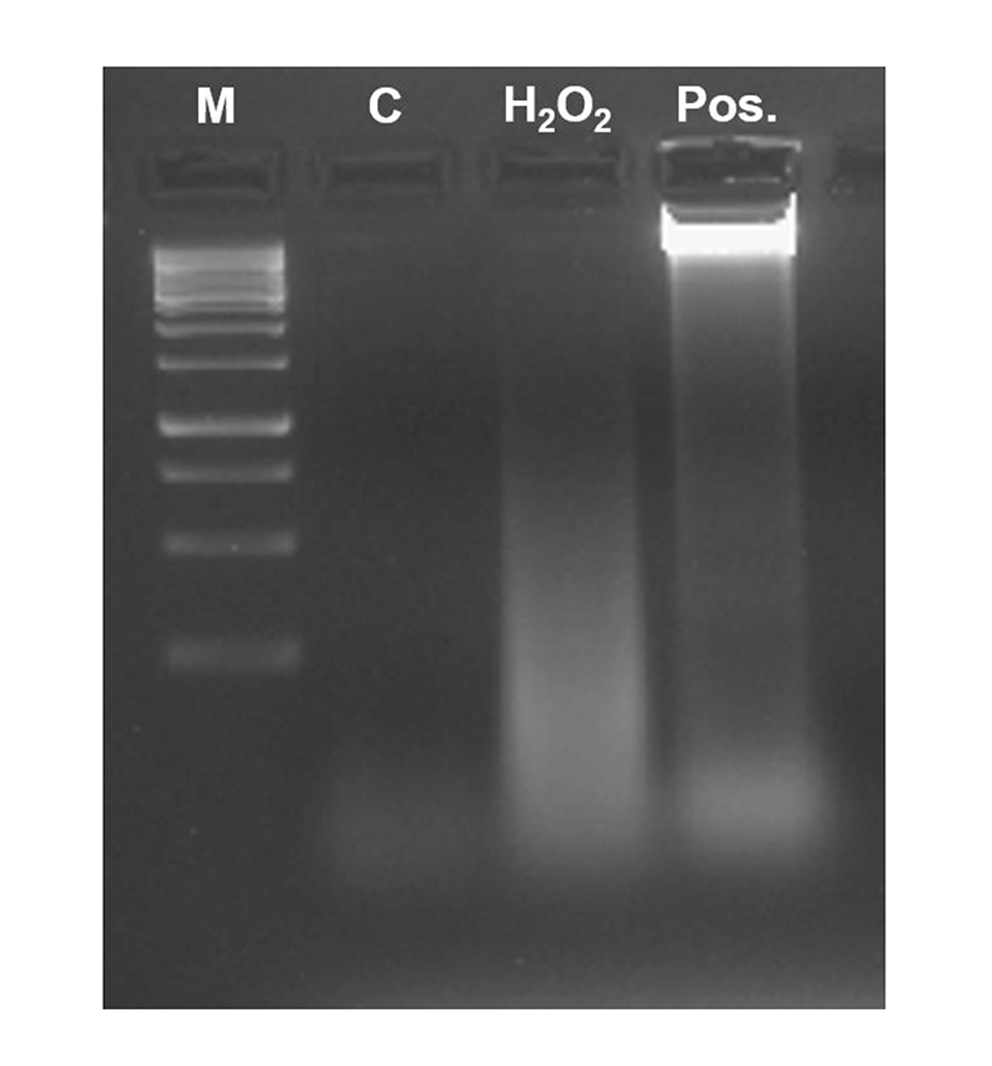

Supplement: Supplementary file 2 — Supplementary file2 (TIF 3598 KB) [file 441_2021_3445_MOESM2_ESM.tif]

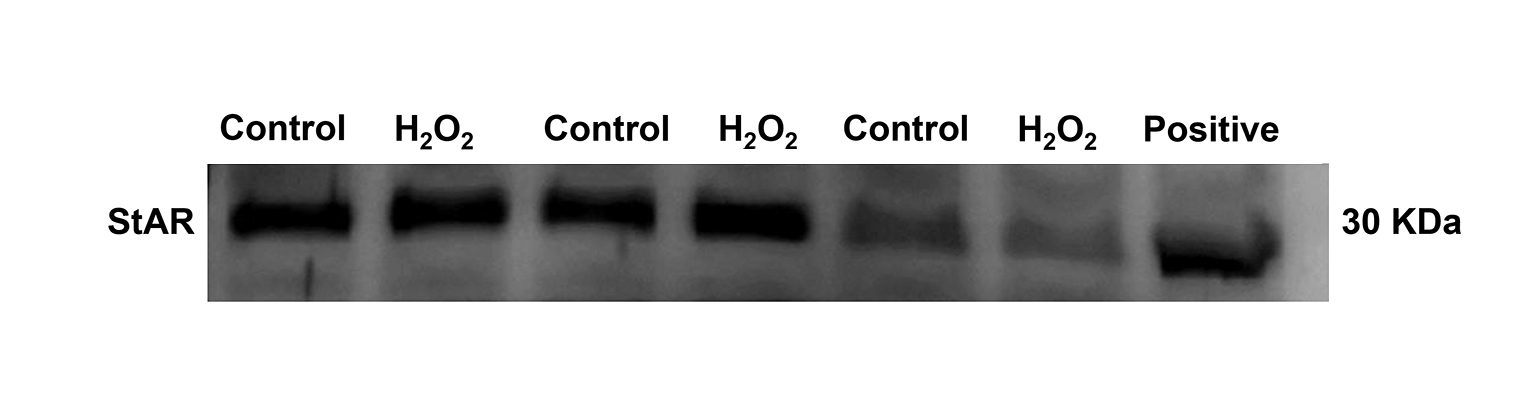

Supplement: Supplementary file 3 — Supplementary file3 (TIF 2068 KB) [file 441_2021_3445_MOESM3_ESM.tif]

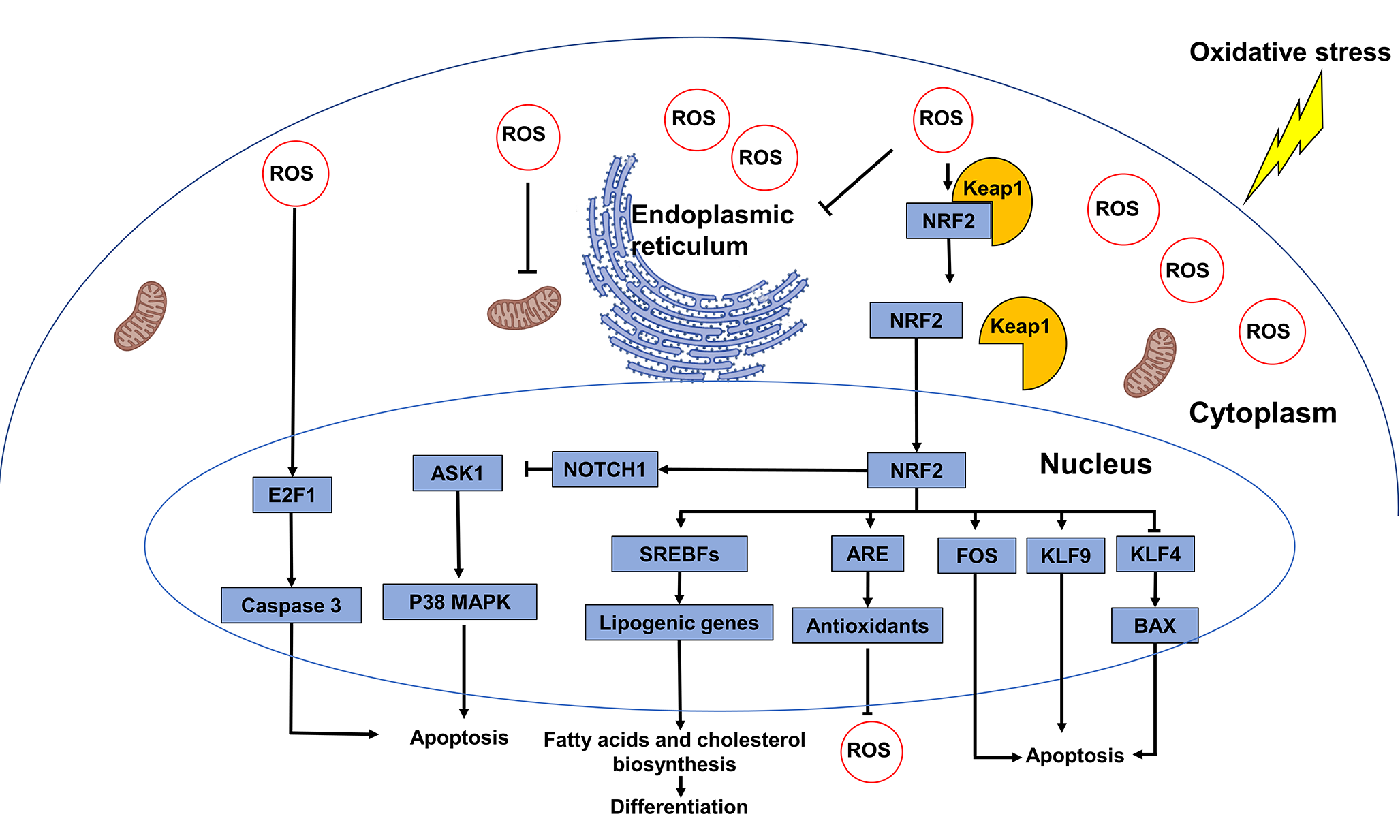

Supplement: Supplementary file 4 — Supplementary file4 (TIF 1111 KB) [file 441_2021_3445_MOESM4_ESM.tif]
